# Combined Cell Therapy in the Treatment of Neurological Disorders

**DOI:** 10.3390/biomedicines8120613

**Published:** 2020-12-15

**Authors:** Daria D. Namestnikova, Elvira A. Cherkashova, Kirill K. Sukhinich, Ilya L. Gubskiy, Georgy E. Leonov, Leonid V. Gubsky, Alexander G. Majouga, Konstantin N. Yarygin

**Affiliations:** 1 Department of Neurology, Neurosurgery and Medical Genetics, Pirogov Russian National Research Medical University of the Ministry of Healthcare of Russian Federation, 117997 Moscow, Russia; golerus@gmail.com (G.E.L.); gubskii@mail.ru (L.V.G.); 2 Radiology and Clinical Physiology Scientific Research Center, Federal Center of Brain Research and Neurotechnologies of the Federal Medical Biological Agency of Russian Federation, 117997 Moscow, Russia; tchere@yandex.ru (E.A.C.); gubskiy.ilya@gmail.com (I.L.G.); 3 Laboratory of Problems of Regeneration, Koltzov Institute of Developmental Biology of the Russian Academy of Sciences, 119334 Moscow, Russia; transpl@hotmail.com; 4 D. Mendeleev University of Chemical Technology of Russia, 125047 Moscow, Russia; Alexander.majouga@gmail.com; 5 Laboratory of Biomedical Nanomaterials, National University of Science and Technology “MISIS”, 119049 Moscow, Russia; 6 Faculty of Chemistry, Lomonosov Moscow State University, 119991 Moscow, Russia; 7 Laboratory of Cell Biology, Orekhovich Institute of Biomedical Chemistry of the Russian Academy of Sciences, 119435 Moscow, Russia; kyarygin@yandex.ru; 8 Russian Medical Academy of Continuous Professional Education of the Ministry of Healthcare of Russian Federation, 125993 Moscow, Russia

**Keywords:** neurological diseases, cell therapy, combined therapy, binary therapy, transplantation

## Abstract

Cell therapy of neurological diseases is gaining momentum. Various types of stem/progenitor cells and their derivatives have shown positive therapeutic results in animal models of neurological disorders and in clinical trials. Each tested cell type proved to have its advantages and flaws and unique cellular and molecular mechanism of action, prompting the idea to test combined transplantation of two or more types of cells (combined cell therapy). This review summarizes the results of combined cell therapy of neurological pathologies reported up to this point. The number of papers describing experimental studies or clinical trials addressing this subject is still limited. However, its successful application to the treatment of neurological pathologies including stroke, spinal cord injury, neurodegenerative diseases, Duchenne muscular dystrophy, and retinal degeneration has been reported in both experimental and clinical studies. The advantages of combined cell therapy can be realized by simple summation of beneficial effects of different cells. Alternatively, one kind of cells can support the survival and functioning of the other by enhancing the formation of optimum environment or immunomodulation. No significant adverse events were reported. Combined cell therapy is a promising approach for the treatment of neurological disorders, but further research needs to be conducted.

## 1. Introduction

Diseases of the central and peripheral nervous system affect millions of people and constitute one of the major causes of death and disability worldwide. Many neurological disorders lead to irreversible damage of neural tissue, lifelong neurological deficit, and decreased quality of life [1]. Currently available treatment and rehabilitation methods are still insufficient, not least due to the limited regeneration capacity of the nervous system. In recent years, transplantation of various types of autologous or allogeneic stem/progenitor cells, including embryonic and induced pluripotent stem cells, hematopoietic, bone marrow mononuclear and mesenchymal/stromal stem cells, vascular stem/progenitor cells, neural stem/progenitor cells, and others, as well as more differentiated progeny of stem cells, has shown promising therapeutic results in the animal models of several common neurological diseases and in clinical trials [2,3,4]. Each cell type has been assigned certain mechanisms of action, which are at least partly different from the mechanisms of action of other cells. For example, cells of the neural lineage, such as neural stem and progenitor/precursor cells, olfactory ensheathing cells, oligodendrocyte and motoneuron progenitor cells are supposed to provide replacement and neuroprotective effects, integrate into the host neural network, promote endogenous neurogenesis, stimulate brain plasticity and synaptic remodeling [5,6,7]. Mesenchymal stem cells have proven paracrine activity, ability to migrate towards inflamed sites, and stimulate progenitor cell proliferation and angiogenesis [8,9]. Moreover, mesenchymal stem cells have anti-inflammatory, immunomodulatory, and immunosuppressive properties, and are reported to conceal themselves from the host immune system (meticulously reviewed in [10]). Transplantation of vascular progenitor cells may contribute to the recovery of neurological function due to induction of angiogenesis and protection of the neurovascular niche [11]. All the mentioned cell types were reported to readily respond to chemokines generated at the sites of tissue injury and inflammation by moving towards those sites along the gradient of chemokine concentration [10,12,13]. However, the exact mechanisms of stem cells’ action determining their positive therapeutic effects remain not fully understood.

Nevertheless, the accumulated evidence concerning the effects of various cell types and the underlying mechanisms of action supports the idea to test combined transplantation of several stem/progenitor cell species in order to obtain synergic effect and to increase the efficacy of therapy. Currently this approach is rapidly developing, though the number of papers on this subject is still limited. Published papers describe combined cell therapy of such common socially significant neurological disorders of adult age and adolescence as stroke, spinal cord injury, neurodegenerative diseases, Duchenne muscular dystrophy, and retinal degeneration. In this review, we summarized the available results and analyzed the advantages of combined cell therapy of the mentioned neurological pathologies in animal studies and first clinical trials.

## 2. Ischemic Stroke

Ischemic stroke is one of the leading causes of death and disability worldwide [14]. Nowadays, only intravenous thrombolysis with tissue plasminogen activator and mechanical thrombectomy are the approved therapeutic methods in the acute period of stroke [15]. However, even after the timely performed reperfusion therapy many neurons and glial cells die or become dysfunctional and patients suffer from residual functional deficits. Besides, there is no effective treatment in subacute, chronic, and late chronic stage of stroke except neurorehabilitation [16] and prevention of recurrent stroke [17]. Cell, especially stem/progenitor cell transplantation holds great promise as an effective strategy for stroke treatment according to the results of numerous animal studies and clinical trials [18,19,20]. Various types of stem cells including embryonic stem cells, induced pluripotent and reprogrammed stem cells, bone marrow mononuclear cells, hematopoietic stem cells, mesenchymal stem cells, neural stem/precursor cells, olfactory ensheathing cells, vascular progenitors, and others have been studied and demonstrated therapeutic benefits (for review see [18,21]). However, these studies did not reveal the most effective single stem cell type, as well as optimal cell dose, administration routes, and transplantation time window. With the goal to enhance the effectiveness of treatment, cotransplantation of different stem/progenitor cell types was explored in several studies.

### 2.1. Results of Animal Studies

Li et al. tested the hypothesis that enhancing both neural and vascular recovery could be more effective than neural repair alone [22]. They performed combined transplantation of the murine fetal neural progenitor (NPC) and vascular progenitor (VPC) cells into the peri-infarct area 24 h after transient middle cerebral artery occlusion (MCAO) in rats. Within the 14-day period, combined transplantation produced better functional recovery and faster reduction of stroke volume than the NPC administration alone. The survival of both neural and vascular graft was also higher in case of cotransplantation. Moreover, the presence of VPC stimulated migration of NPC in the brain toward the infarct area and promoted differentiation and maturation of neuronal cells. In the study carried out by Nakagomi et al. [23], intracerebral transplantation of murine adult neural stem cells (NSC) and endothelial cells into immunodeficient mice enhanced animal survival and proliferation of NSC and their accelerated neuronal differentiation. Mice treated with NSC and endothelial cells showed improved cortical function. Taken together, these data indicate that reconstruction of the elements of the “neurovascular niche” can be an effective therapeutic strategy in the treatment of stroke.

Synergistic effects were reported after combined transplantation of NSC and mesenchymal stem cells (MSC) as well. Hosseini et al. [24] injected rat bone marrow MSC and, after 7 days, rat fetal NSC intraventricularly in MCAO rats. In 28 days after stroke combined cell administration promoted better functional recovery and reduction of brain lesion compared to single cell therapy. The positive therapeutic effects of combined transplantation of MSC and endothelial progenitor cells (EPC) in cerebral ischemia were also observed and the results were scrutinized in the meta-analysis paper published by Sun et al. [25]. In this work it was demonstrated that the use of combined cell therapy resulted in significant decrease of neurological impairment compared to the administration of MSC only. The cerebral infarction volume was lower in the cotransplantation group in comparison with the EPC- and MSC-alone groups. The levels of brain derived neurotrophic factor in the brain of the cotransplantation group animals were higher than in the EPC-alone group animals [25].

### 2.2. Results of Clinical Trials

Joint transplantation of human fetal neural stem/progenitor cells (NSPC) and umbilical cord MSC was tested in eight patients with ischemic stroke within the territories of the middle or anterior cerebral arteries [26]. The patients who were treated with the combination of MSC intravenously and NSPC together with MSC delivered through the cerebellomedullary cistern demonstrated superior improvements compared to the patients who received intravenous infusions of MSC only as evidenced by the National Institutes of Health Stroke Scale (NIHSS), the Barthel index (BI), and the modified Rankin Scale (mRS) scores. Described clinical trials have shown relative safety and feasibility of combined stem cell therapy of stroke in humans, however larger samples, longer follow-ups, and control groups are still needed.

## 3. Spinal Cord Injury

Spinal cord injury (SCI) is a damage of the spinal cord that leads to disorganization of sensory, motor, and autonomic function, affects patient’s physical and psychological condition, impairs quality of life and limits social independence [27]. The estimated global incidence is 40–80 cases per one million population and up to 90% of cases happen because of traumatic incidents [28]. In the initial phase of acute SCI the main damage to the neural tissue is inflicted by fractures or dislocation of the vertebrae, post-traumatic microhemorrhages, overstretching and contusion of spinal cord, cell membrane destruction and axonal damage. Later a variety of pathophysiological processes causing secondary medullary damage is induced, including inflammation, homeostasis deregulation, ionic imbalance, glutamate release, increased apoptosis, and necrosis [29].

Many pathophysiological mechanisms of SCI are well understood, but presently there is no effective way to treat such patients. Commonly used neurorestorative strategies include early reduction and fixation of the injured vertebrae, extramedullary and intramedullary decompression, neuroprotection and prevention of further neurodegeneration in order to minimize the incurable consequences of SCI [30]. Cell therapy is one of the promising treatments for acute and subacute SCI. There have been many basic, preclinical, and clinical studies demonstrating beneficial effects of the transplantation of different types of stem/progenitor cells to treat this condition (reviewed by Jin et al. [31] and Vismara et al. [32]). Functional and partial structural restoration of spinal cord was observed after administration of embryonic stem cells, induced pluripotent stem cells, neural stem cells, olfactory ensheathing cells, and Schwann cells. Immunomodulatory effects and trophic support have been described after the mesenchymal stem cells administration [33,34].

Recent advances in the therapy with single stem cell type are promising. However, full recovery after complete spinal cord injury (grade A according to American Spinal Injury Association Scale (ASIA)) still could not be achieved. The prospective strategies to improve functional restoration of the spinal cord could include the transplantation of stem/progenitor cells together with implantation of various biomaterials or scaffolds made of them [31,35,36] or combined administration of different stem/progenitor cell types.

### 3.1. Results of Animal Studies

Combined transplantation of different cell types has been described in several basic studies on animal SCI models. Erceg et al. assessed the therapeutic potential of cotransplantation of oligodendrocytes and motoneuron progenitor cells derived from human embryonic stem cells directly into the spinal cord of rats in acute phase after complete spinal cord transection [37]. Both types of injected cells survived, migrated, and differentiated to mature oligodendrocytes and neurons at the lesion site. First signs of locomotor function improvement were seen 3 weeks after transplantation. The recovery of the motor-evoked potential (MEP) measured by electrophysiology was observed later, 40 days after injection. The authors supposed that recoveries of MEP can be attributed to the reconnection of the axons above and below the lesion site and the contribution of oligodendrocytes. Synergistic effect of cotransplantation of murine embryonic stem cell-derived motor neurons (ESMN) and olfactory ensheathing cells (OEC) was observed in the study by Salehi et al. [38]. OEC and ESMN were transplanted into the lesion site 9 days after modeling of SCI contusion in rats. Significant recovery of hindlimb function was shown in cell transplantation groups compared to control groups 4 weeks after transplantation. Though there was no difference in functional recovery between combined and single cell therapy groups, tissue sparing, myelin ratio, and the survival rate of ESMN were higher after cotransplantation. The authors hypothesized that OEC supports the survival of ESMN through released neurotrophic factors and cell adhesion molecules. In another study Park et al. [39] injected human bone marrow MSC intravenously immediately after SCI contusion modeling in rats and transplanted human fetal NSC directly into the damaged area of spinal cord 1 week later. In the cotransplantation group recovery of hindlimb motor function was observed at the end of the 6-week study period, however there were no significant differences compared to the group with direct administration of NSC only. The absence of the synergic effect of combined transplantation may be the result of different ways of administration and their separation in time.

### 3.2. Results of Clinical Trials

Therapeutic effects of combined cell therapy of SCI were also estimated in patients with SCI during clinical trials. One prospective randomized double-blind clinical study [40] aimed to compare the efficacy of cell therapy with human fetal OEC only, fetal Schwan cells only (SC), or fetal OEC together with fetal SC in a small cohort of patients. It was shown that cell transplantation was well tolerated, and only mild adverse effects were observed (fever in one patient with OEC transplantation). All patients who received cell therapy showed functional improvement, as evidenced by electrophysiological tests. Another report [41] describes the effects of the combined intrathecal administration of allogeneic umbilical cord blood CD34+ cells and placenta derived MSC 5 and 8 months after traumatic spinal cord injury, supplemented with additional intravenous administration 6 months later. No adverse immunological reactions or graft-versus-host disease were revealed. An enhancement of muscle strength, increased sensation in various dermatomes, improved urologic, sexual, and bowel functions, decreased pain were noted during the observation period of 2 years. Moviglia et al. [42] published a case report about transplantation of three different types of stem cell into the patients with chronic low cervical and thoracic spinal cord injury (ASIA grade A). First, autologous bone marrow mononuclear cells (MNC) were injected into the artery feeding the disrupted area of the spinal cord. In 18 days, specific spinal cord effector T cells were transplanted intravenously, and it was followed by another targeted intra-arterial administration, this time of transdifferentiated from MSC autologous neural stem cells. The author did not detect autoimmune reactions or adverse events. Functional improvement was observed in five patients (evolved from ASIA grade A to D) and two patients remained in the same condition (ASIA grade A), but exhibited motor and sensitive improvements.

The described clinical studies proved that combined cell therapy of SCI is safe and in general has beneficial effects in patients. However, its advantage over cell therapy employing just one type of cell was not clearly demonstrated in clinical trials.

## 4. Neurodegenerative Diseases

Neurodegenerative diseases are characterized by the progressive loss of neurons in the brain, resulting in cognitive impairment, motor neuron dysfunction and other neurological problems. Alzheimer’s disease and Parkinson’s disease are the most common representatives of this group of pathologies [43]. Neurodegenerative diseases affect millions of people worldwide and the risk of being affected increases dramatically with age. Cell therapy as a potential treatment for neurodegenerative diseases has been studied for more than 40 years and generally is considered effective [44,45]. In this context, NSC were shown to be promising candidates for cell replacement therapy, while cotransplantation of NSC with OEC improved long-term survival of transplanted NSC by providing trophic support [46].

### Results of Animal Studies

Srivastava et al. investigated therapeutic effects of the cotransplantation of rat fetal OEC and NPC in the rat model of neurodegenerative disease induced by kainic acid [47]. Kainic acid causes selective neuronal death in the rat brain in a way similar to excitotoxic action of glutamate in Alzheimer’s disease [48]. Twelve weeks after intracerebral administration of OEC and NPC significant recovery in learning and memory was shown in the group with combined transplantation of the two cell types in comparison with single cell type treatment. Additionally, the expression of choline acetyltransferase and restoration of the cholinergic receptor binding with specific radioligand [3H]-QNB were significantly higher in the cotransplantation group. The authors suggested that OEC were able to synthesize neurotrophic factors that improve the long-term survival of transplanted NPC. This hypothesis was supported by the in vitro studies that had shown that NPC cocultured with OEC had higher differentiation capacity and functional activity compared to NPC cultured alone.

Another paper reported rat OEC cotransplantation with rat fetal ventral mesencephalic cells (VMC) in the 6-hydroxydopamine rat model of Parkinson’s disease [49]. In this study animals receiving combined intracerebral injection of OEC and VMC demonstrated significantly better functional restoration compared to control groups according to the results of neurobehavioral tests performed 12 weeks after cell cotransplantation. The number of the tyrosine hydroxylase-positive cells and the density of tyrosine hydroxylase-positive fibers in the striatum increased significantly in animals transplanted with OEC and VMC.

Taken together, these data confirm that OEC can support the viability of transplanted NPC and provide better functional restoration in neurodegenerative diseases. However, further investigations and clinical trials are required.

## 5. Duchenne Muscular Dystrophy

Muscular dystrophies are a group of mostly congenital neuromuscular diseases characterized by progressive weakness and muscle degeneration. They also affect the central nervous system, causing speech and vision impairment, developmental delay, and seizures [50,51]. The most common disease of this group is the Duchenne muscular dystrophy [52]. Its global prevalence is 7.1 cases per 100,000 males and 2.8 cases per 100,000 in the general population [53]. The Duchenne muscular dystrophy is caused by spontaneous or inherited mutations in the dystrophin gene residing in the short arm of chromosome X at the Xp21.2 locus. The majority of patients have a deletion (≈68%) or duplication (≈11%) of one or more exons in the dystrophin gene, however in 20% of patients point mutations were also found [54]. These mutations can occur anywhere in the gene and lead to the absence of the encoded dystrophin protein which in turn leads to myofibers degeneration during contraction, necrosis, inflammation, and finally to muscle atrophy and progressive muscular weakness. The onset of the disease is usually seen between 3 and 5 years of age and worsens over time. Dystrophin is also believed to have a role in brain development [55].

There is still no curative treatment for the disease. Current standard therapy is generally aimed to delay the onset of disabling symptoms and to maximize the quality of life [56]. The new advances in the Duchenne muscular dystrophy experimental treatment include exon skipping and gene and stem cell therapy [57]. The transplantation of allogeneic [58,59,60,61] and iPS-derived [62,63] myoblasts and myogenic precursor/progenitor cells, muscle satellite cells [64], mesoangioblasts [65], and mesenchymal stem cells [66,67,68] in both preclinical experiments and clinical trials has been reported and limited positive effects demonstrated. Inadequate cell engraftment, their low proliferative potential and chronic systemic, and local inflammation in the affected muscles were named the main reasons for the insufficient efficacy of single cell type cell therapy of the disease [69,70].

### Results of Clinical Trials

There is only one report describing the efficacy of combined cell therapy in Duchenne muscular dystrophy. Klimczak et al. performed combined transplantation of bone marrow-derived MSC and skeletal muscle-derived stem/progenitor cells (SM-SPC) [70] obtained from HLA-matched related healthy donors in three patients with Duchenne muscular dystrophy. Cells were injected directly into the biceps brachii and gastrocnemius muscles with short-time immunosuppression using tacrolimus. The efficacy of therapy was confirmed by an increase in motor unit parameters according to electroneuromyography study during the 6-month follow-up of patients. The decrease in blood creatine kinase levels and normalized profile of proinflammatory cytokines in the blood serum was also observed. Muscle biopsies taken from the patients and subsequent PCR analysis showed decrease in the content of adipose and fibrous tissue in the skeletal muscles, and an increased level of dystrophin expression. Thus, cell administration directly into the affected muscle, but not into the nervous tissue have shown to slow down the progression of muscular dystrophy. There was no evidence of side effects in the described study. Both applied cell populations were reported to fuse with degenerating skeletal muscle fibers triggering their recovery. It was suggested that trophic, paracrine, and immunomodulatory activity of MSC may support the proregenerative potential and engraftment of SM-SPC.

## 6. Retinal Degenerative Diseases

The retina is a thin layer of neural cells that lines the inner back surface of the eyeball and is actually a part of the central nervous system [71]. Retinal degeneration diseases comprise different pathologies that cause damage or malfunction of retinal cells and can lead to vision loss of varying degrees up to complete blindness. Among the most common retinal degenerative diseases are retinitis pigmentosa and age-related macular degeneration [72]. Currently available therapeutic methods are not specific and not effective enough [73,74]. Stem cell transplantation to the retina can be one of the promising approaches according to the results of animal studies and phase I-II clinical investigations [75].

In recent studies it has been shown that cell therapy employing just one cell type, namely NSC [76,77], MSC [78], or retinal progenitor cells [79] was safe and induced beneficial effects including immunomodulation and improvement of retinal morphology and function resulting in the delay of retinal degeneration. However, the use of one type of stem cell may have some limitations. For example, NSC alone were effective during a small treatment window and triggered gliosis [80], while MSC differentiation into retinal cells was critically difficult [81].

### Results of Animal Studies

Currently, combined transplantation of different types of cells for the treatment of retinal degeneration was attempted only in animal models. Zhai et al. [80] demonstrated that injection of rat OEC and rat fetal NSC into the subretinal space of rats with inherited retinal degeneration (the royal college of surgeons (RCS) rats) leads to more significant electrophysiological improvement of b-wave amplitudes in electroretinogram compared to rats receiving injections of either OEC or NSC. Cotransplantation of NSC with OEC activated endogenous stem cells in the retina soon after transplantation decreased gliosis and ensured better photoreceptor survival. It was also demonstrated that in vitro OEC increased NSC migration ability, enhanced their stemness, and reduced gliotic tendency, possibly revealing the basic mechanisms underlying the reinforcement of the therapeutic effect in the case of combined cell transplantation. In another paper Qu et al. [81] reported functional improvement of injured photoreceptors after transplantation of human retinal progenitor cells (HRPC) and human bone marrow-derived MSC into the subretinal space of RCS rats. Besides, HRPC differentiated into photoreceptors more effectively when injected in combination with MSC. MSC transplantation also enhanced survival and migration of HRPC in vivo, which might be related to MSC-induced immunosuppression. Furthermore, activation of microglia and the gliosis of Müller cells were more effectively suppressed in combined transplantation. Therefore, cotransplantation cell therapy has a potential to become an effective strategy for the treatment of retinal degenerative diseases and clinical investigations are needed.

## 7. Discussion

The number of publications devoted to the studies of coadministration of different stem/progenitor cell types in animals with experimentally modeled neurological disorders is still very limited (summarized in Table 1). It applies even more to I/II phase clinical trials dedicated to the safety and feasibility of cell therapy of neurological diseases combining different cell types (summarized in Table 2). Despite the limited number of publications on this subject, the already available data suggest that cotransplantation can have significant advantages over single cell type therapy. The advantages include better functional recovery, enhanced survival, proliferation, migration, and differentiation of transplanted cells.

In most studies the authors focused on the efficacy of cotransplantation, while the mechanisms underlying synergistic effects of combined cell therapy were examined less extensively. Those mechanisms can be diverse. Transplanted cells can act independently and in this case their effects are simply aggregated. On the other hand, the effects of different cell types may be interdependent due to direct or indirect cell interactions. According to the above-cited publications, one kind of transplanted cells can support the survival and functioning of the other cell type. Indeed, better neural cell graft survival was observed in case of its combined administration with vascular/endothelial progenitor cells [22,23] and olfactory ensheathing cells [38]. Most probably, this could be the result of the neurovascular niche restoration and formation of the optimum microenvironment [23,82] combined with the neuroprotective and neuroregenerative effects of OEC [83,84]. Better engraftment of NSC/NPC may promote functional recovery through direct cellular replacement mechanism [85,86] or the induction of repair mechanisms mediated by the paracrine delivery of trophic factors, immunomodulation, stimulation of neurogenesis and angiogenesis, and enhancement of neuroplasticity (well reviewed in [5]). NSC/NPC demonstrate paracrine activity of their own, and it can be combined with paracrine effects of other cell types, for example MSC [24]. Cotransplantation of MSC was also reported to promote enhanced survival of retinal progenitor cells [81], skeletal muscle-derived stem/progenitor cells [70] and to prevent microglia activation and gliosis in the retina [81] and fibrosis in the muscular tissue [70]. Most likely these effects can be attributed to remarkable immunomodulatory/anti-inflammatory activity of MSC [9,87,88] and their angiogenic, antiapoptotic, protective, and antioxidative properties, which are mediated mainly through paracrine mechanisms (reviewed in [89]) and partly by direct cell-to-cell communications [90]. Moreover, MSC allegedly enhance cell viability through forming cell-to-cell contacts for transfer of mitochondria to damaged cells [90,91,92].

In a few studies on spinal cord injury the benefits of combined cell therapy have not been confirmed. This could be the result of the severity of injury or wrong selection of cell types, delivery route, and transplantation timing.

Importantly, in all reviewed studies no significant adverse events in animals and humans were reported. However, it is worth noting that human studies were single case reports or phase I/II clinical trials with a small number of patients and many more investigations with bigger sample size, randomization, and placebo control are needed before combined cell therapy of neurological disorders can be transferred into clinical practice. Since a number of experimental and clinical studies reviewed here demonstrated enhanced efficacy of combined cell therapy in a number of neurological disorders in the adult animals and humans, there is a good reason to believe that it may be also effective in neonatal brain pathologies and in children with cerebral palsy and autism spectrum disorders, as suggested in the comprehensive review by Paton et al. [93].

In general, combined cell therapy of neurological disorders may be a prospective treatment approach. However, there are still many questions to answer. It is evident that further research needs to be conducted.

## Figures and Tables

**Table 1:** Summary of the results of combined cell therapy in animal studies. EC—endothelial cells; ESC—embryonic stem cells; ESMN—embryonic stem cell-derived motorneurons; EPC—endothelial progenitor cells; HRPC—human retinal progenitor cells; IV—intravenous; IVT—intraventricular; IS—intraspinal; MCAO—middle cerebral artery occlusion; MPC—motoneuron progenitor cells; MSC—mesenchymal stem cells; NPC—neural progenitor cells; NSC—neural stem cells; OEC—olfactory ensheathing cells; OPC—oligodendrocyte progenitor; SCI—spinal cord injury; VMC—ventral mesencephalic cells; VPC—vascular progenitor cells.

Study	Pathology	Species	Cell Types	Way of Delivery	Immunosuppression	Time of Delivery	Advantages of Combined over Single Cell Therapy
Li et al. [22]	MCAO stroke model	Rats	Fetal murine NPC + VPC	IC sequential injections of both types of cells;	Cyclosporine A	24 h after MCAO	Functional recovery; stroke volume reduction; neural and vascular graft better survival; stimulated migration, differentiation, and maturation of NPC
Nakagomi et al. [23]	MCAO stroke model	Mice	Murine adult NSC + EC	IC sequential injections of both types of cells	The recipients were immunosuppressive mice	7 days after MCAO	Improved cortical function; enhanced survival, proliferation and differentiation of NSC
Hosseini et al. [24]	MCAO stroke model	Rats	Rat bone marrow MSC + fetal NSC	IVT	No	MSC 1 day after and NSC 7 days after MCAO	Better functional recovery; reduction of stroke volume
Erceg [37]	SCI complete transection model	Rats	Human ESC-derived OPC + MPC	IS	No	Immediately after SCI	Electrophysiological improvement
Salehi et al. [38]	SCI contusion model	Rats	Murine ESMN + OEC	IS	Cyclosporine A	9 days after SCI	Functional recovery, but no significant difference compared to single cell type therapy; enhanced ESMN survival; better tissue sparing and myelin ratio
Park et al. [39]	SCI contusion model	Rats	Human bone marrow MSC + fetal NSC	IV + IS	No	MSC IV immediately after and NSC IS 7 days after SCI	Functional recovery, but no significant difference compared to single cell type therapy
Srivastava et al. [47]	Kainic acid-induced model of Alzheimer’s disease	Rats	Rat adult OEC+ fetal NPC	IC	No	4 weeks post-lesioning	Learning and memory recovery; enhanced expression of choline acetyltransferase and cholinergic receptors binding
Agrawal et al. [49]	6-hydroxydopamine—lesioned model of Parkinson’s disease	Rats	Rat adult OEC + fetal VMC	IC	No	5 weeks post-lesioning	Functional recovery; more tyrosine hydroxylase-positive cells and higher density of tyrosine hydroxylase-positive fibers
Zhai et al. [80]	Model of inherited retinal degeneration (Royal College of Surgeons (RCS) rats)	Rats	Rat adult OEC + fetal NSC	Into subretinal space	The recipients were RCS rats	4 weeks postnatally	Electrophysiological improvement according to electroretinogram; activation of endogenous retinal stem cells; reduced gliosis; enhanced NSC migration; better photoreceptor survival
Qu et al. [81]	Model of inherited retinal degeneration (Royal College of Surgeons (RCS) rats)	Rats	Human HRPC + human bone marrow MSC	Into subretinal space	The recipients were RCS rats	3 weeks postnatally	Electrophysiological improvement according to electroretinogram; better survival, migration, and differentiation of HRPC; reduced gliosis

**Table 2:** Summary of the results of combined cell therapy and clinical trials. ENMG—electroneuromyography; IA—intraarterial; IV—intravenous; IVT—intraventricular; IS—intraspinal; IT—intrathecal; MSC—mesenchymal stem cells; NSC—neural stem cells; NSPC—neural stem/progenitor cells; OEC—olfactory ensheathing cells; SC—Schwann cells; SCI—spinal cord injury; SM-SPC-skeletal muscle-derived stem/progenitor cells.

Study	Pathology	Phases of Clinical Research	Cell Types	Way of Delivery	Time of Delivery	Advantages of Combined over Single Cell Therapy
Qiao et al. [26]	Ischemic stroke	Phase I/II eight patients no control group/placebo control; no randomization; 2-year follow-up	Human fetal NSPC + umbilical cord MSC	MSC IV multiple times + NSPC through the cerebellomedullary cistern one time	Acute phase post-stroke	Better NIHSS, BI, mRS scores, but no control group
Klimczak et al. [70]	Dushenne muscular dystrophy	Phase I/II three patients no control group/placebo control; no randomization; 6-month follow-up	Human allogenic bone marrow MSC + SM-SPC	Direct IM	Patients between 11 and 22 years old	Electrophysiological improvement according to ENMG; decreased blood creatine kinase level; normalized profile of proinflammatory cytokines; decrease in the content of adipose and fibrous tissue in muscles; increased level of dystrophin expression
Moviglia et al. [42]	SCI	Case reports eight patients	Human autologous bone marrow MSC + spinal cord specific effector T cells + autologous NSC	MSC IA + 18 days later effector T cells IV + NSC IA	Chronic phase post injury	Increased ASIA score grade, but no control group
Ichim et al. [41]	SCI	Case report one patient	Human allogenic umbilical cord blood CD34 + placental derived MSC	IT	Within first year after injury	Functional improvement, but no control group
Chen et al. [40]	SCI	Phase I/II 28 patients prospective randomized double-blind no placebo 12-month follow-up	Human fetal OEC + SC	IS	At least 12 months after injury	Functional recovery, but no significant difference compared to single cell type therapy
